# Smoking and Glioma Risk: Evidence From a Meta-Analysis of 25 Observational Studies: Erratum

**DOI:** 10.1097/MD.0000000000026977

**Published:** 2021-08-20

In the article “Smoking and Glioma Risk: Evidence From a Meta-Analysis of 25 Observational Studies”,^[1]^ which appears in Volume 95, Issue 2 of *Medicine*, several reference citations appeared incorrectly in the text. This has been updated in the text and the corrections are described here. In the sentence “Five studies were eligible for the dose–response analysis of duration of smoking with glioma risk.^22,26,28,29,34^” the references should be 21, 25, 27, 28,33. In the sentence “There are 4 studies providing the sufficient data required for dose effect of smoking intensity.^17,26–28^” the references should be 17,25–27. In the sentence “Five studies investigated a dose–risk relationship between number of pack-years smoking and glioma.^22,26,28,29,34^” the references should be 21, 25, 27, 28, 33.
